# Supplementary figures and images for: The core outer junction protein CFAP77 connects A- and B-tubules within doublet microtubules of cilia and flagella
Source: PLoS Biol. 2025 Oct 21;23(10):e3003442. doi: 10.1371/journal.pbio.3003442 (PMC12551952; doi:10.1371/journal.pbio.3003442)

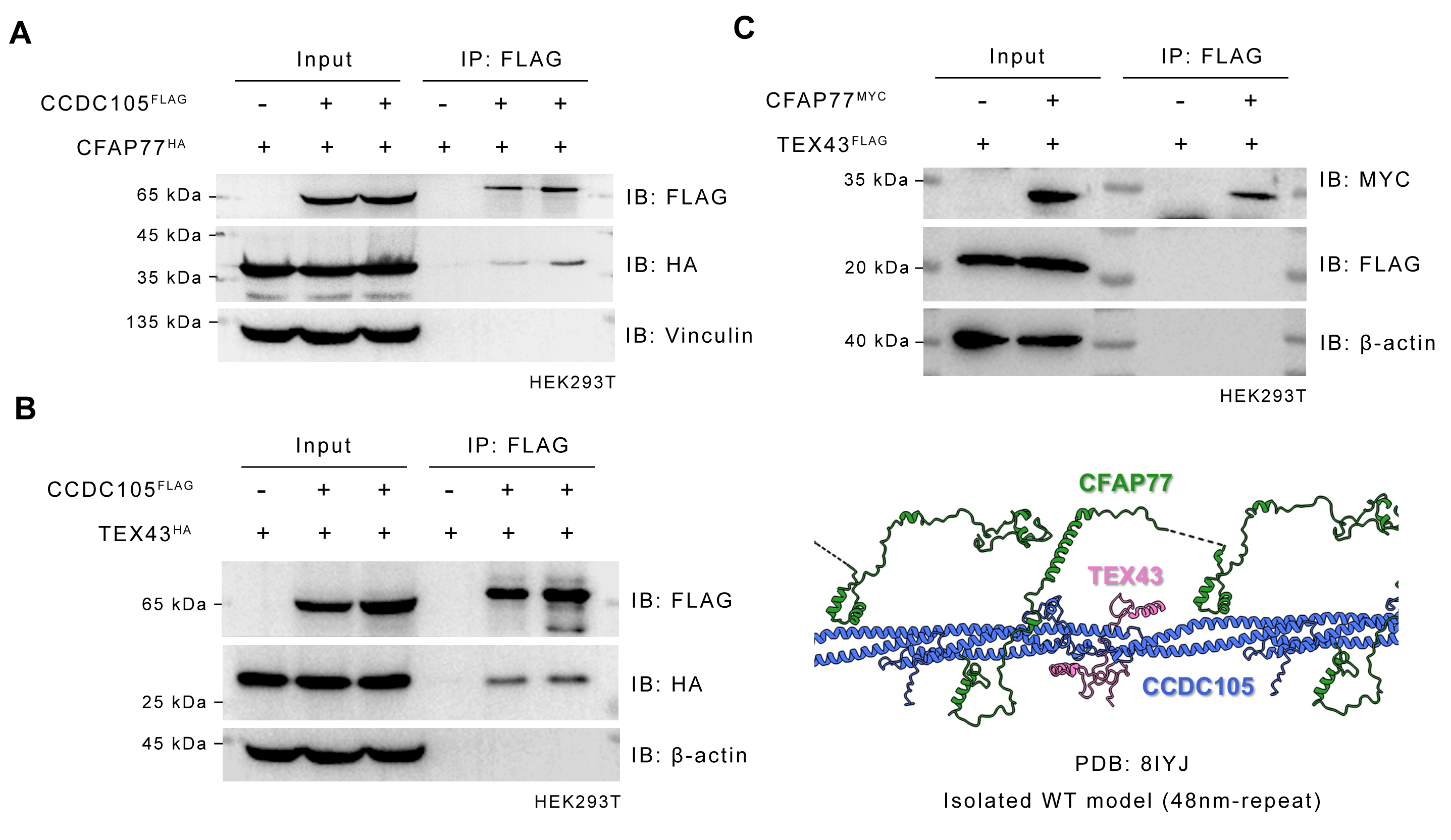

Supplement: S1 Fig — (A) HA-tagged CFAP77 could be immunoprecipitated by FLAG-tagged CCDC105 in HEK293T cells. Vinculin served as the internal control. (B) HA-tagged TEX43 could also be immunoprecipitated by FLAG-tagged CCDC105 in HEK293T cells. β-actin served as the internal control. (C) No interaction between MYC-tagged CFAP77 and FLAG-tagged TEX43 in HEK293T cells. β-actin served as the internal control. These coimmunoprecipitation results were consistent with the structural data showing that CCDC105 interacts with both CFAP77 and TEX43 but that there is no interaction between CFAP77 and TEX43. Raw blot images can be found in S1 Raw Images. (TIF) [file pbio.3003442.s001.tif]

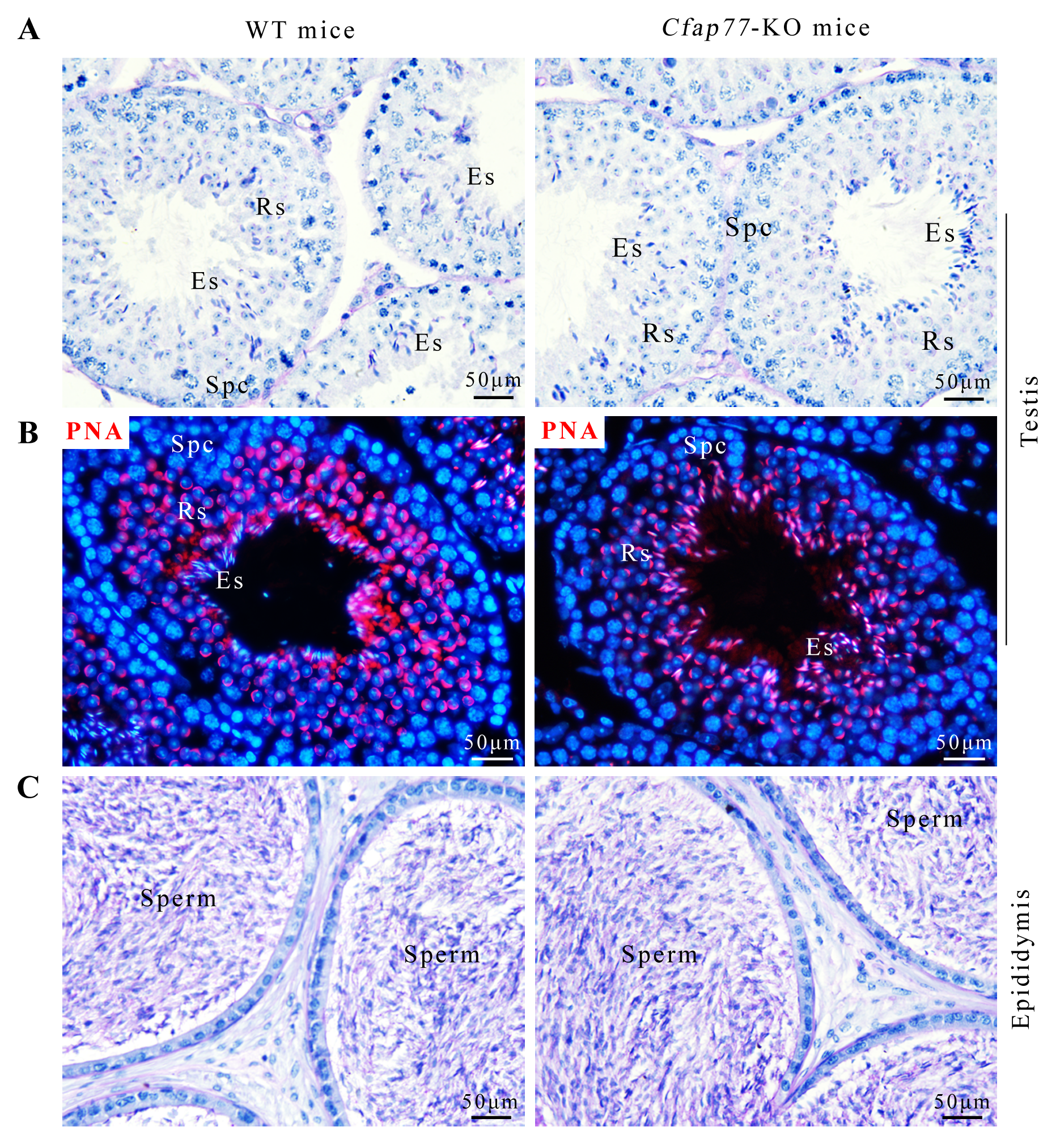

Supplement: S2 Fig — (A) Representative histological morphology of testis sections from Cfap77-KO mice and WT mice determined by periodic acid-schiff (PAS) staining. Spc, spermatocytes; Rs, round spermatids; Es, elongating spermatids. Scale bars, 50 μm. (B) Staining of PNA (peanut agglutinin)-TRITC to reveal acrosomal formation in testis sections from Cfap77-KO mice and WT mice. Nuclei were stained with DAPI. (C) Representative histological morphology of the cauda epididymis of Cfap77-KO mice and WT mice, as determined by haematoxylin and eosin (H&E) staining. (TIF) [file pbio.3003442.s002.tif]

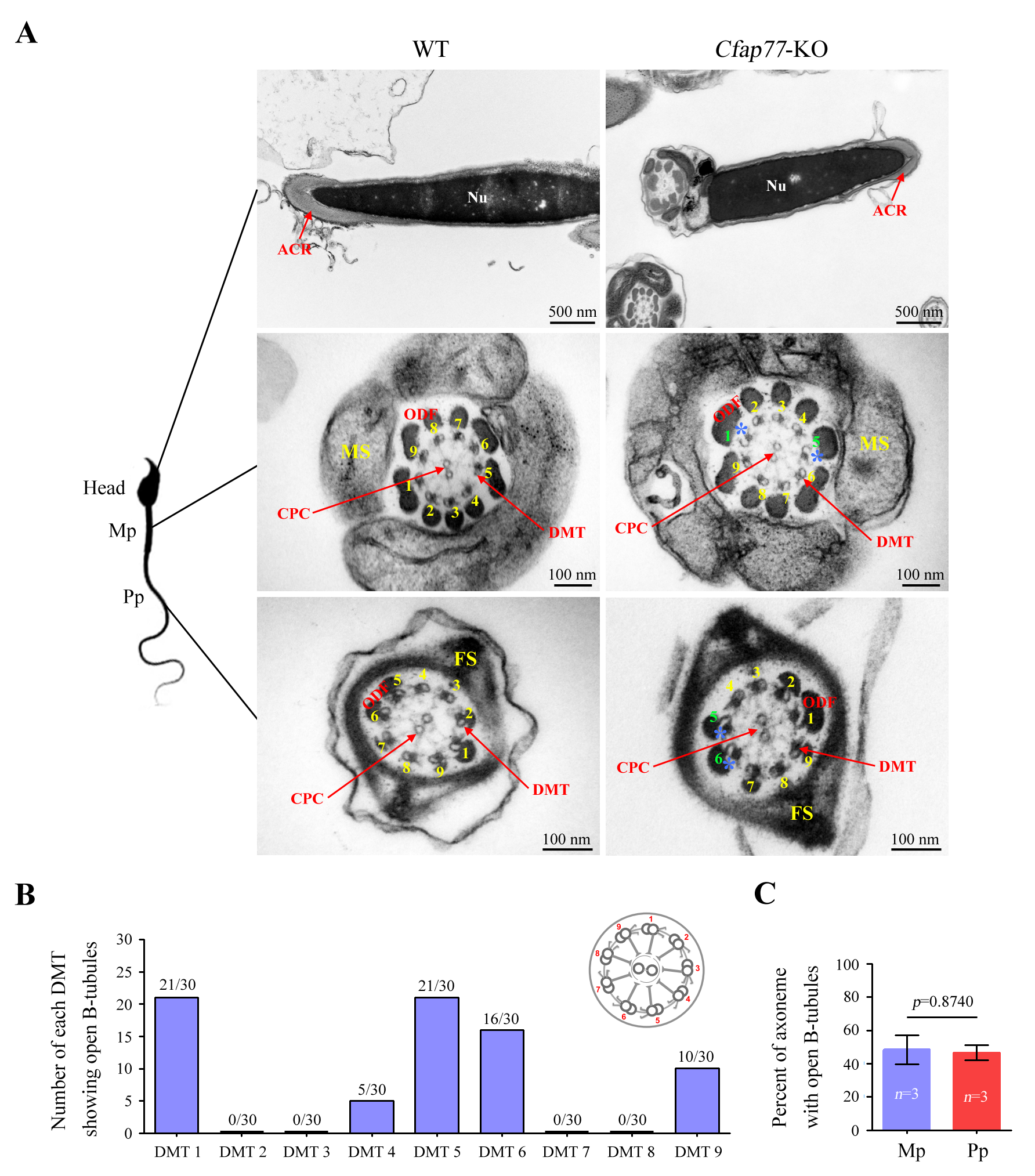

Supplement: S3 Fig — (A) Transmission electron microscopy analysis of acrosome (ACR), mid-piece (Mp) and principal piece (Pp) of the tails of WT mice and Cfap77-KO mice. Nu, nucleus; MS, mitochondrial sheath; FS, fibrous sheath; CPC, central pair complex; DMT, doublet microtubule; ODF, outer dense fibre. Scale bars, 100 nm or 500 nm. (B) Number of each DMT (1–9) exhibiting the open B-tubules. A total of 30 axonemes with open DMT B-tubules in Cfap77-KO sperm were counted. (C) The percentage of axonemes with open DMT B-tubules was calculated between the Mp and Pp of sperm flagella in Cfap77-KO mice. Student t test; error bars represent the SEM (n = 3). The data underlying the graphs shown in the figure can be found in S1 Data. (TIF) [file pbio.3003442.s003.tif]

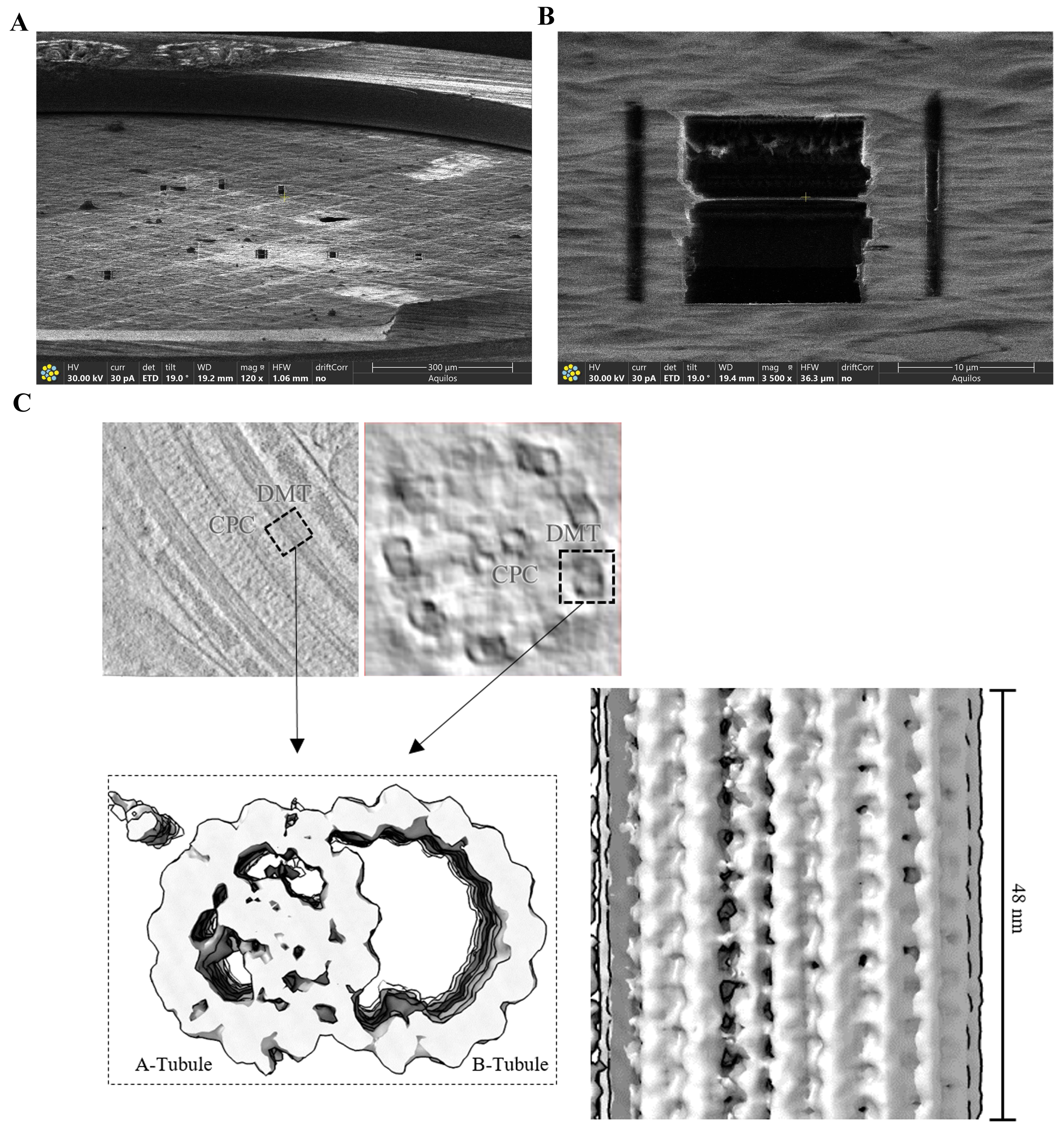

Supplement: S4 Fig — (A) Inspection of frozen sperm on the grid. (B) Inspection of frozen sperm on the grid after FIB milling. The thin lamellae were used for data collection. (C) In-cell structural determination of sperm axonemal DMTs from Cfap77-KO mice. Side and transverse sectional views of mouse sperm axonemes are shown in the tomogram slices. (TIF) [file pbio.3003442.s004.tif]

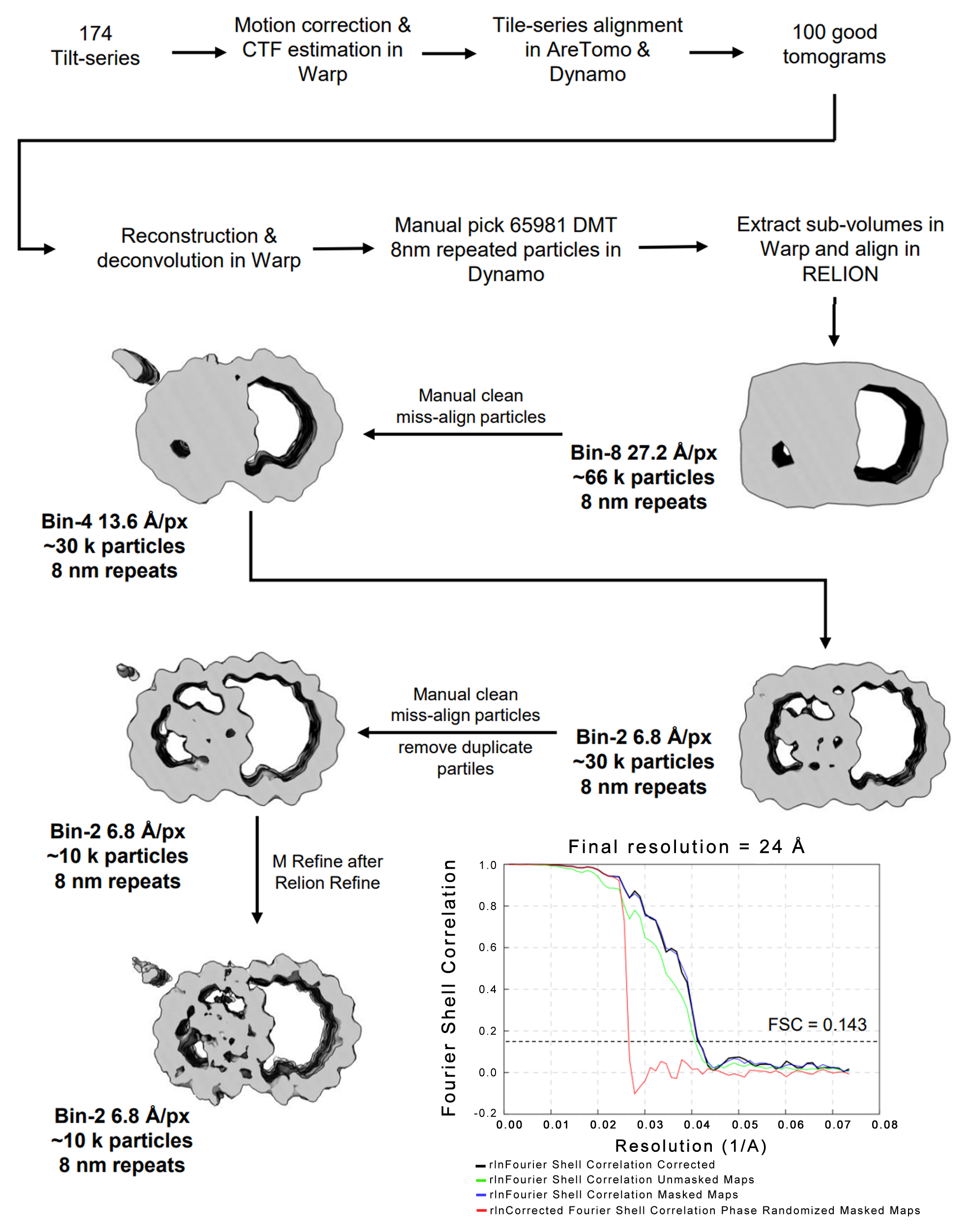

Supplement: S5 Fig — The pixel sizes at different binning levels are indicated in angstroms per pixel (Å/px for short). The half-map Fourier shell correlation (FSC) plot is shown. (TIF) [file pbio.3003442.s005.tif]

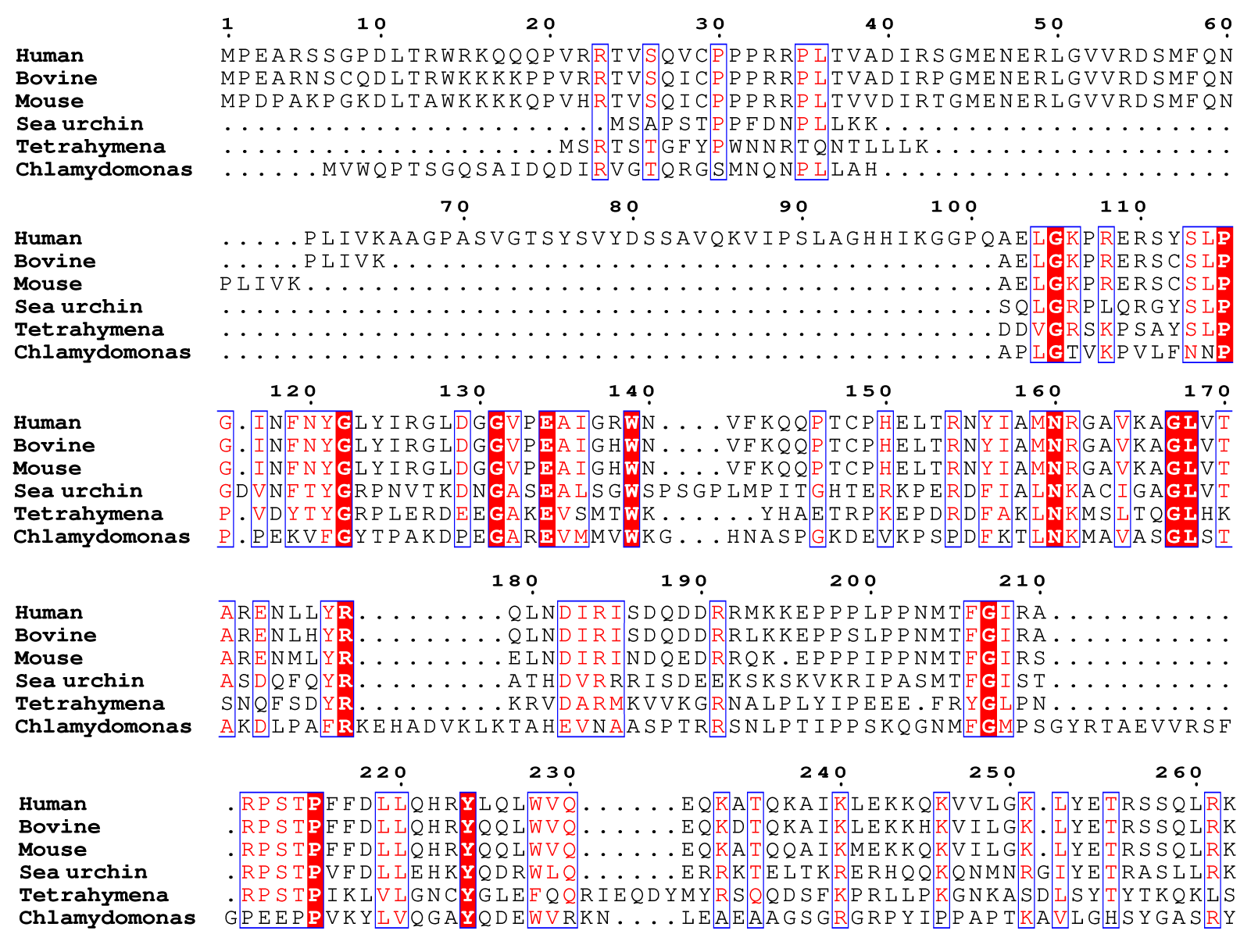

Supplement: S6 Fig — Using the ClustalW and ESPript websites, we revealed that CFAP77 was remarkably conserved among humans, bovines, mice, sea urchins, Tetrahymena, and Chlamydomonas. The amino acid sequences of CFAP77 were obtained from UniProt: human (Q6ZQR2), bovine (A0A3Q1LJD6), mouse (A0A087WRI3), sea urchin (A0A7M7TG06), Tetrahymena (Q22WR6), and Chlamydomonas (A8IB22). (TIF) [file pbio.3003442.s006.tif]

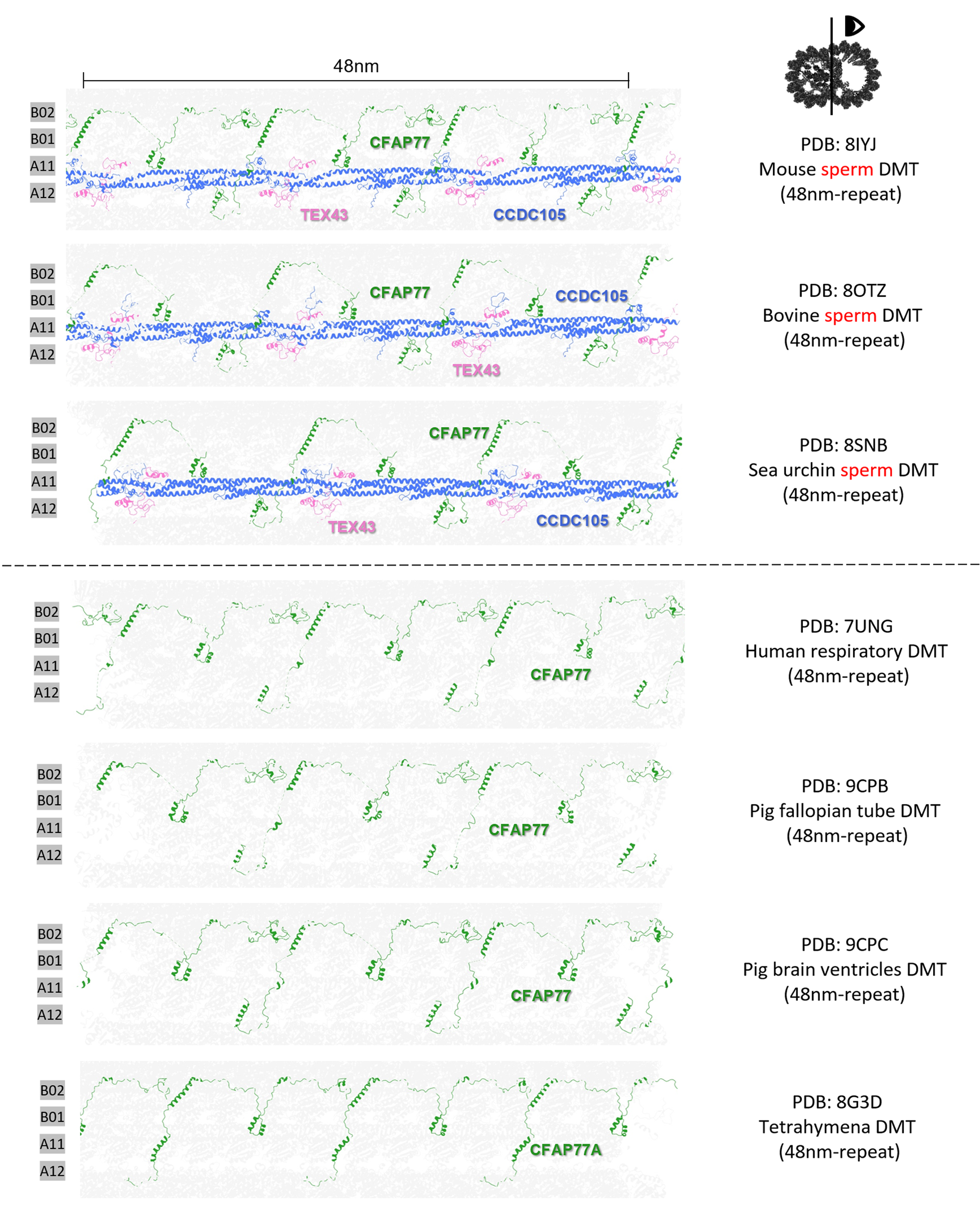

Supplement: S7 Fig — Structural models of the CFAP77-CCDC105-TEX43 subcomplex or CFAP77 at the OJ regions of axonemes from various species and tissues are presented, with a vertical section showing a 48 nm length of the DMTs. From top to bottom, the models include mouse sperm (PDB: 8IYJ), bovine sperm (PDB: 8OTZ), sea urchin sperm (PDB: 8SNB), human respiratory epithelium (PDB: 7UNG), pig fallopian tube (PDB: 9CPB), pig brain ventricles (PDB: 9CPC), and Tetrahymena (PDB: 8G3D). (TIF) [file pbio.3003442.s007.tif]

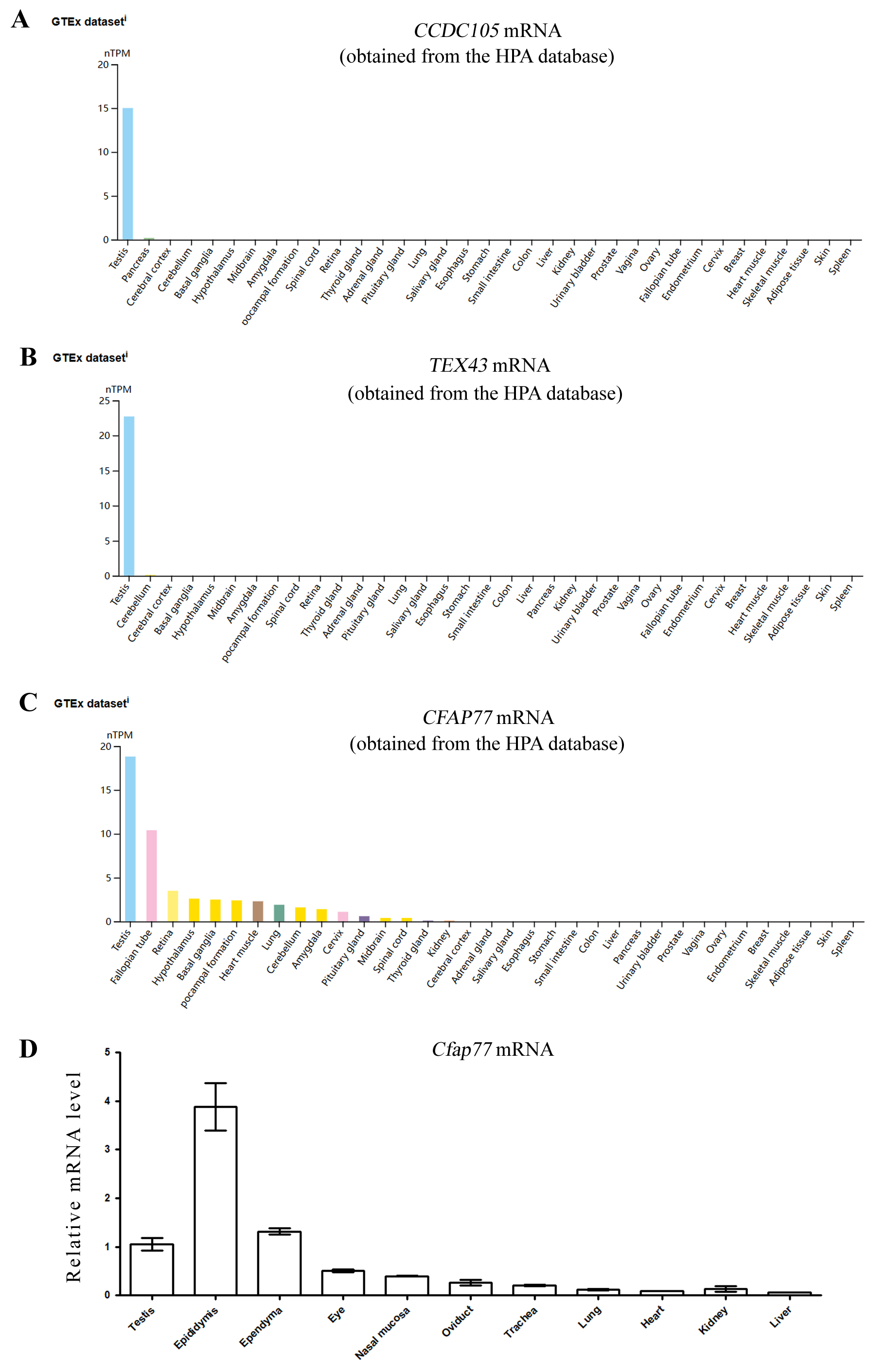

Supplement: S8 Fig — (A) CCDC105 mRNA was restricted to the testes in humans. These data were obtained from the Human Protein Atlas (HPA) database (https://www.proteinatlas.org/ENSG00000160994-CCDC105/tissue). (B) TEX43 mRNA was also restricted to the testes in humans. These data were obtained from the HPA database (https://www.proteinatlas.org/ENSG00000196900-TEX43/tissue). (C)Human CFAP77 mRNA was predominantly expressed in the testes but was also present in other ciliary tissues, including the fallopian tube, retina, brain, and lung. These data were obtained from the HPA database (https://www.proteinatlas.org/ENSG00000188523-CFAP77/tissue). (D) According to the results of quantitative real-time PCR, Cfap77 mRNA was highly expressed in mouse testes and epididymis, but was also expressed in other tissues. Student t test; error bars represent the SEM (n = 3). The data underlying the graphs shown in the figure can be found in S1 Data. (TIF) [file pbio.3003442.s008.tif]

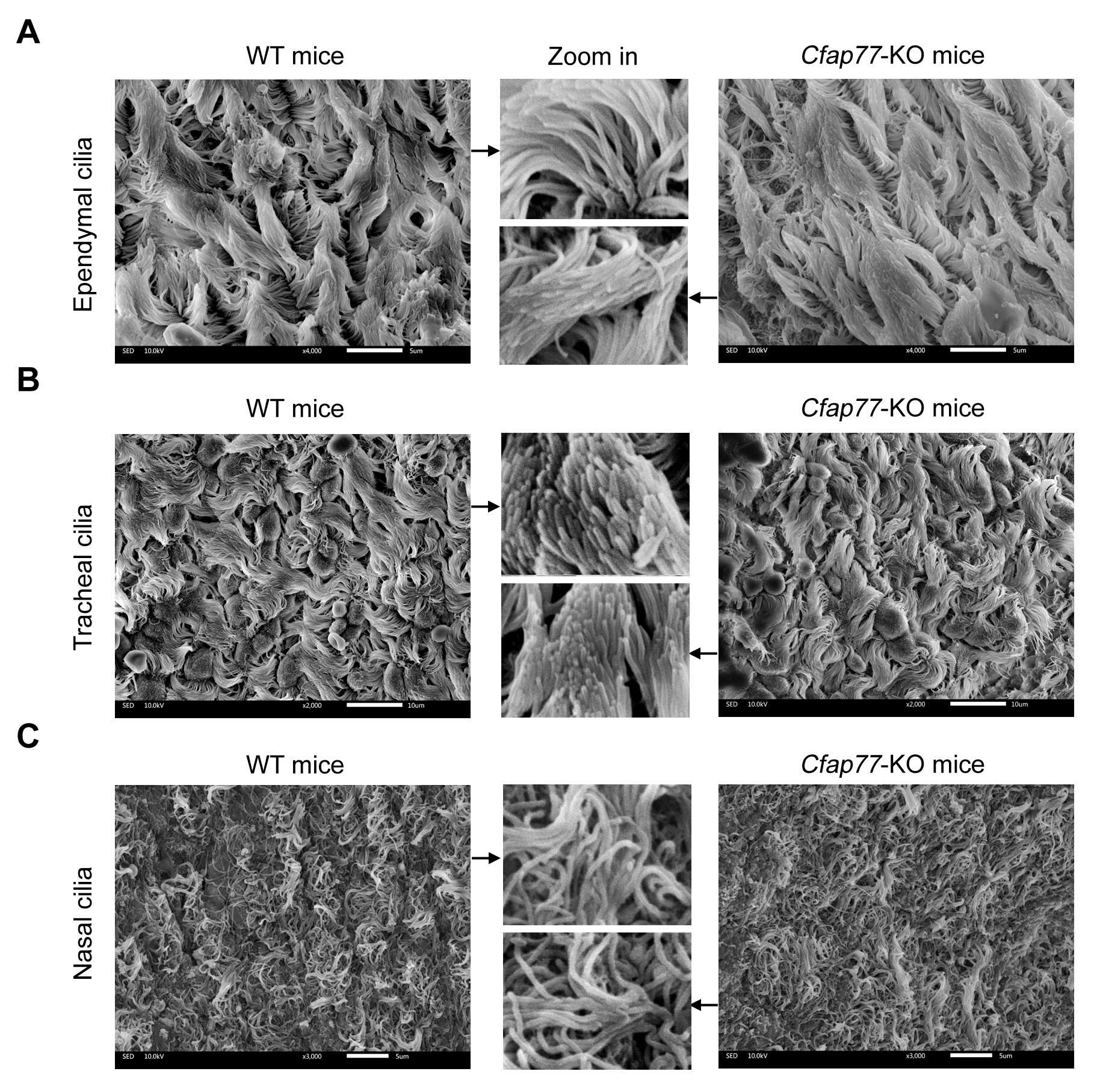

Supplement: S9 Fig — (A) Representative scanning electron microscopy images of brain ependymal cilia from Cfap70-KO mice and WT mice. Scale bar, 5 μm. (B) Representative scanning electron microscopy images of tracheal cilia from Cfap70-KO mice and WT mice. Scale bar, 10 μm. (C) Representative scanning electron microscopy images of nasal cilia from Cfap70-KO mice and WT mice. Scale bar, 5 μm. (TIF) [file pbio.3003442.s009.tif]

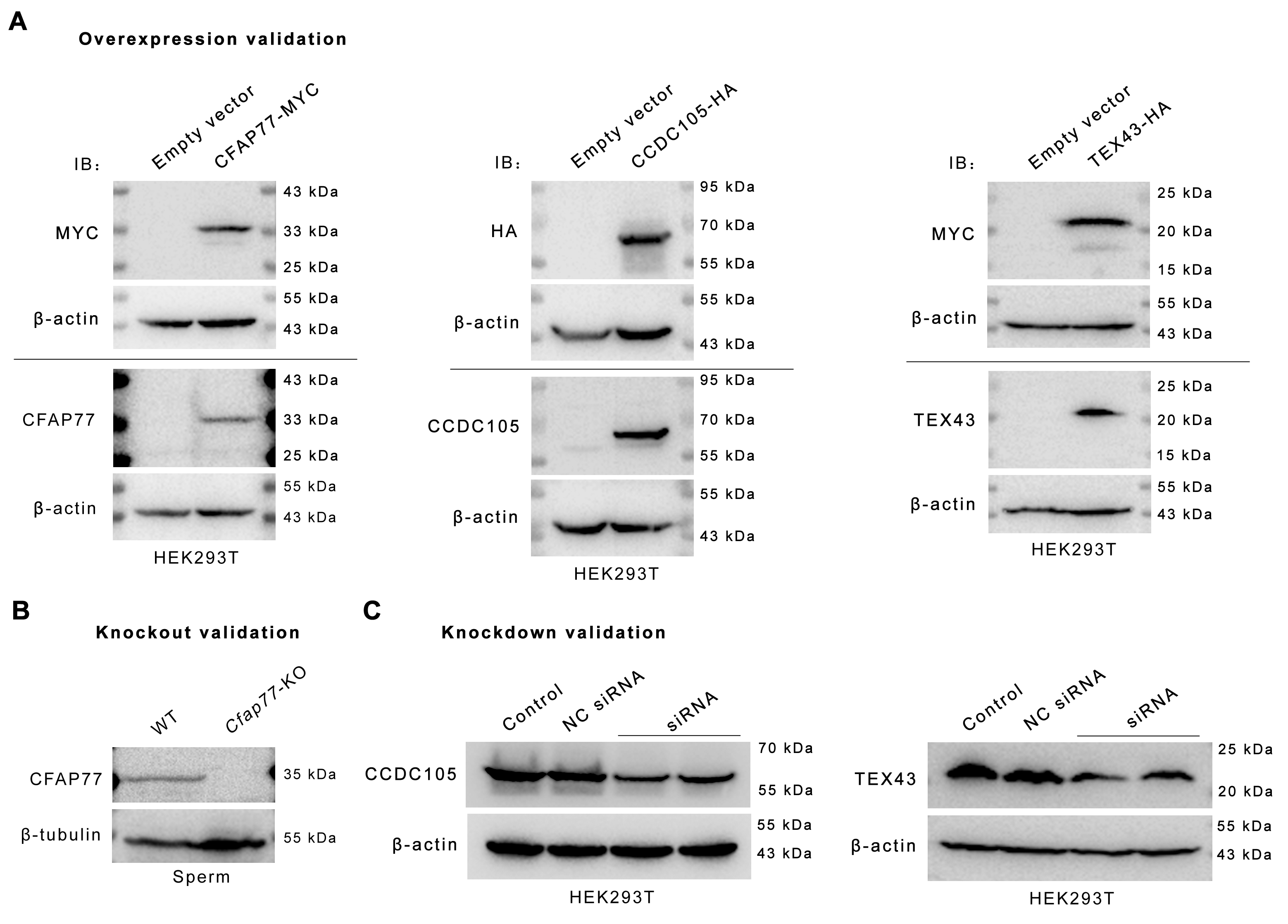

Supplement: S10 Fig — (A) Overexpression verification. HEK293T cells were transfected with CFAP77-, CCDC105-, or TEX43-tagged plasmids; both tag-antibodies and their antibodies recognized a specific band at the same molecular size. (B) Knockout validation. CFAP77 was absent in sperm samples of Cfap77-KO mice. (C) siRNAs targeting Ccdc105 and Tex43 reduced their signals in HEK293T cells transfected with CCDC105- or TEX43-tagged plasmids, respectively. The siRNA sequences were listed as follows. NC siRNA: UUCUCCGAACGUGUCACGUTTACGUGACACGUUCGGAGAATT; siRNA targeting Ccdc105: CGUGUGCUAAGGCCUUGUUTTAACAAGGCCUUAGCACACGTT; siRNA targeting Tex43: GGUGGGACGAUAUUCACUUTTAAGUGAAUAUCGUCCCACCTT. Raw blot images can be found in S1 Raw Images. (TIF) [file pbio.3003442.s010.tif]
