# Supplementary material for: The core outer junction protein CFAP77 connects A- and B-tubules within doublet microtubules of cilia and flagella
Source: PLoS Biol. 2025 Oct 21;23(10):e3003442. doi: 10.1371/journal.pbio.3003442 (PMC12551952; doi:10.1371/journal.pbio.3003442)
Supplement: S1 Table — (DOCX) [file pbio.3003442.s012.docx]

**Table S1. Primers for mouse genotyping.**

| **Primers** | **Sequence** | **Size** |
| --- | --- | --- |
| F1 | 5’-GTCTCGGTTGCTCATTCCTATGT-3’ | Targeted: 484 bp |
| R1 | 5’-GTGTCCCTCATGACCTTCAAGAAA-3’ |  |
| F1 | 5’-GTCTCGGTTGCTCATTCCTATGT-3’ | WT: 827 bp |
| R2 | 5’-CTGTGCCATTTGACTATGGCCTAC-3’ |  |
