# Supplementary material for: The core outer junction protein CFAP77 connects A- and B-tubules within doublet microtubules of cilia and flagella
Source: PLoS Biol. 2025 Oct 21;23(10):e3003442. doi: 10.1371/journal.pbio.3003442 (PMC12551952; doi:10.1371/journal.pbio.3003442)
Supplement: S3 Table — (DOCX) [file pbio.3003442.s014.docx]

**Table S3. Primers for qRT‒PCR.**

| **Target** | **Sequence** | **Size** |
| --- | --- | --- |
| *Ccdc105* | F: TACCGACCTAAGTGTGAGAAGATCC | 195 bp |
|  | R: CATGAGTTCCACAGCTTGCAG |  |
| *Tex43* | F: GAACGTGAATCTACAGCAAGCA | 115 bp |
|  | R: GACGGCTTTCCGATCTTCCC |  |
| *Actb* | F: AACAGTCCGCCTAGAAGCAC | 281 bp |
|  | R: CGTTGACATCCGTAAAGACC |  |
