# Supplementary material for: The core outer junction protein CFAP77 connects A- and B-tubules within doublet microtubules of cilia and flagella
Source: PLoS Biol. 2025 Oct 21;23(10):e3003442. doi: 10.1371/journal.pbio.3003442 (PMC12551952; doi:10.1371/journal.pbio.3003442)
Supplement: S4 Table — (DOCX) [file pbio.3003442.s015.docx]

**Table S4**. Cryo-ET data collection and data processing

| **Data acquisition** | |
| --- | --- |
| Microscope | Titan Krios G2 |
| Voltage (kV) | 300 |
| Detector | Gatan K2 |
| Energy filter | Gatan GIF Quantum, 20 eV |
| Mode | Super resolution |
| Pixel size (Å) | 3.4 |
| Stage tilting angle | -50° - 67° or -66° - 51° |
| Number of images | 39 or 40 |
| Exposure per image (e/Å²) | 3 or 3.5 |
| Exposure per tilt (e/Å²) | 117 to 140 |
| Defocus range (μm) | -1 - -4 |
| Software | SerialEM |
| **Sub tomogram analysis** | |
| Software | RELION-3.1/Warp |
| EMDB ID entry  (composite map) | EMD-63176 |
| Number of tomograms | 100 |
| Number of particles extracted | 65776 |
| Number of final particles | 10020 |
| Symmetry | C1 |
| Resolution (Å) | 24 |
| Map pixel size (Å) | 6.8 |
