# Supplementary material for: The core outer junction protein CFAP77 connects A- and B-tubules within doublet microtubules of cilia and flagella
Source: PLoS Biol. 2025 Oct 21;23(10):e3003442. doi: 10.1371/journal.pbio.3003442 (PMC12551952; doi:10.1371/journal.pbio.3003442)

Fig 1D

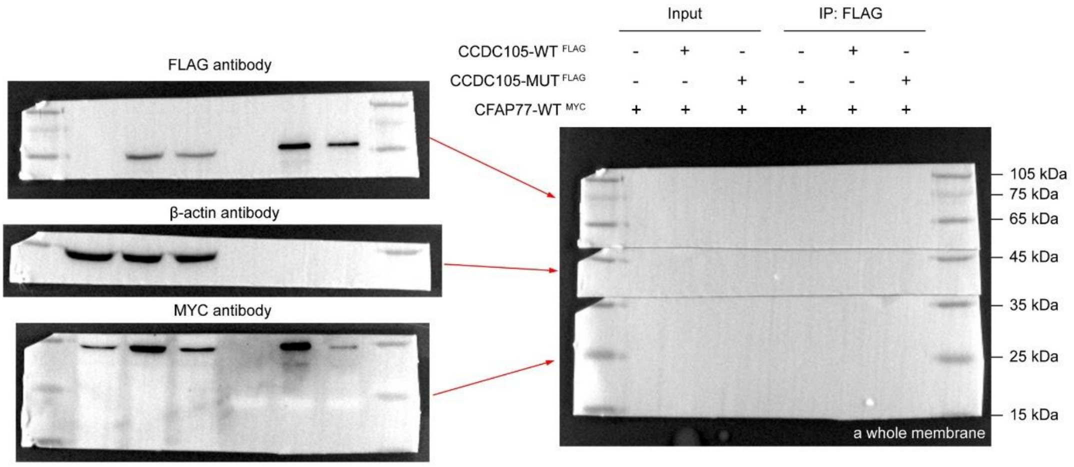

FLAG antibody
β-actin antibody
MYC antibody
Input
IP: FLAG
CCDC105-WT FLAG
CCDC105-MUT FLAG
CFAP77-WT MYC
105 kDa
75 kDa
65 kDa
45 kDa
35 kDa
25 kDa
15 kDa
a whole membrane

Fig 1E

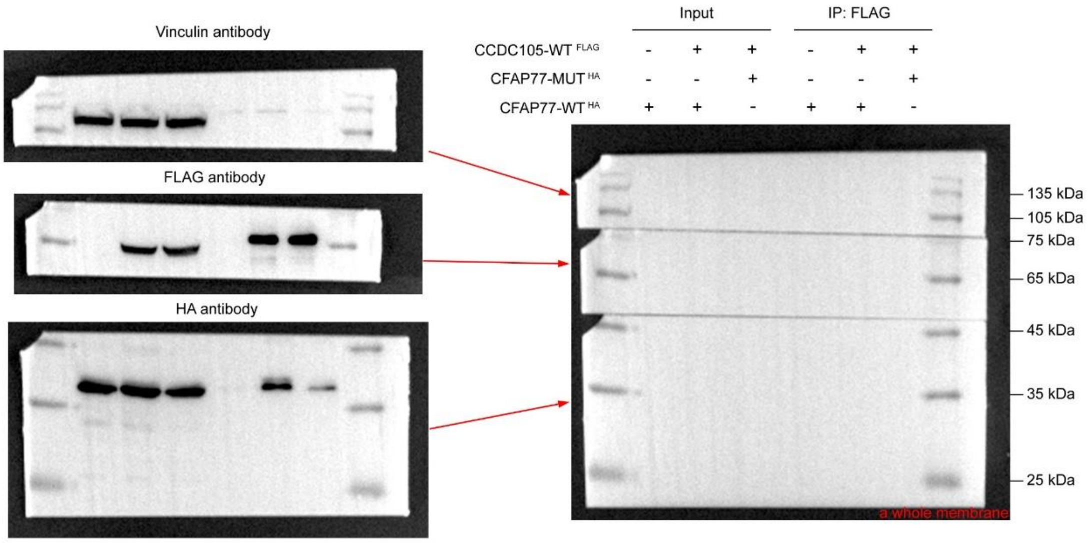

Vinculin antibody
FLAG antibody
HA antibody
Input
IP: FLAG
CCDC105-WT FLAG
CFAP77-MUT HA
CFAP77-WT HA
135 kDa
105 kDa
75 kDa
65 kDa
45 kDa
35 kDa
25 kDa
a whole membrane

Fig 2B

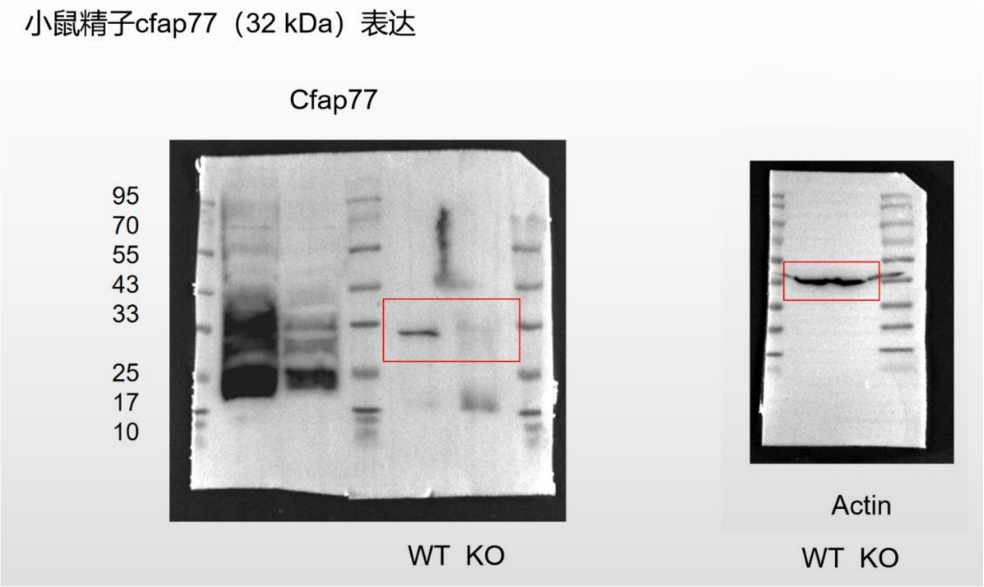
小鼠精子cfap77（32 kDa）表达
Cfap77
95
70
55
43
33
25
17
10
WT KO
Actin
WT KO

Fig 5B

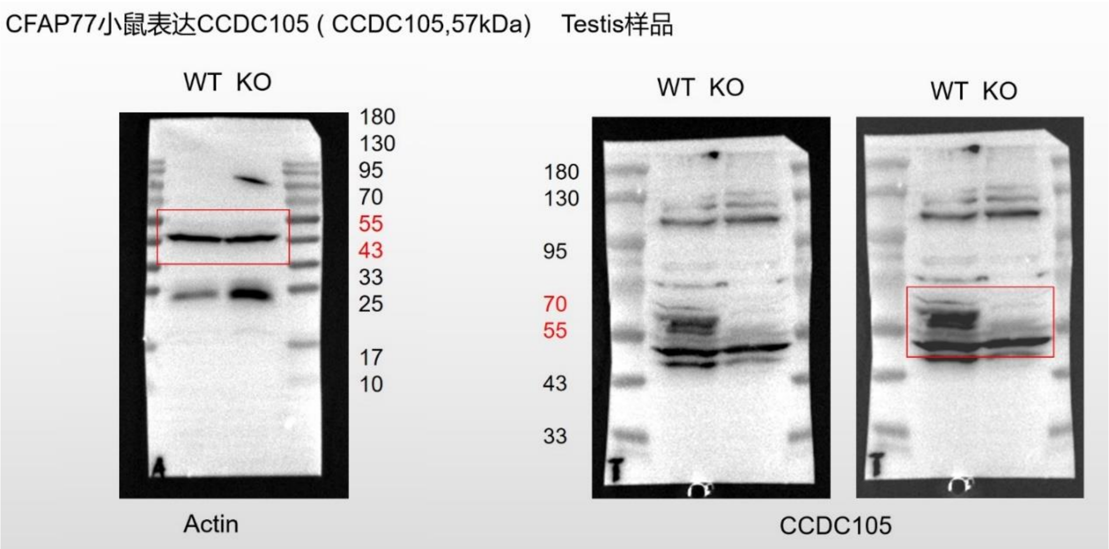
CFAP77小鼠表达CCDC105 (CCDC105,57kDa)　Testis样品
WT KO
180
130
95
70
55
43
33
25
17
10
Actin
WT KO
180
130
95
70
55
43
33
WT KO
CCDC105

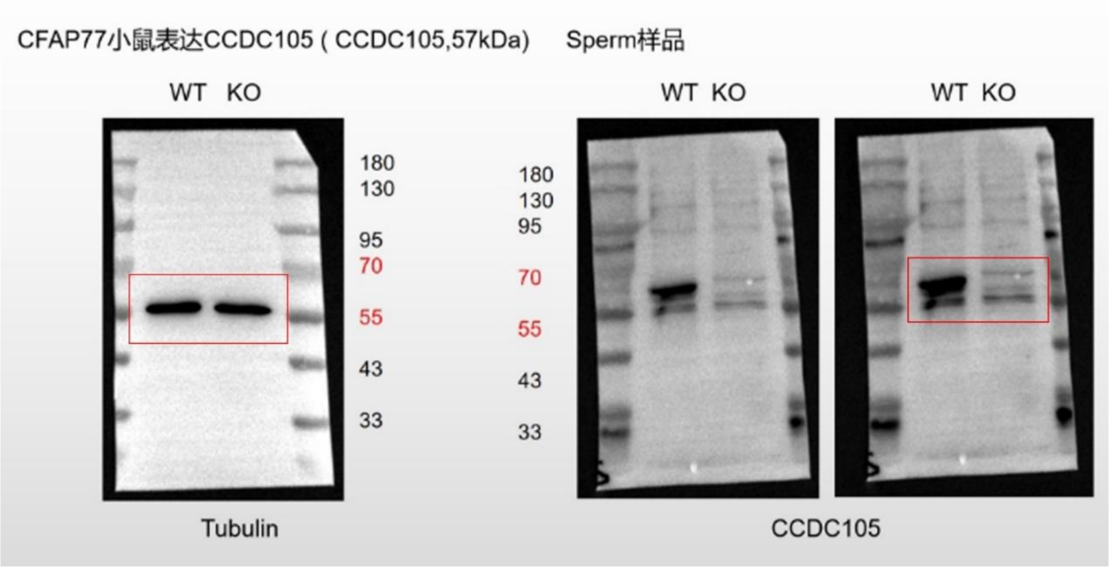
CFAP77小鼠表达CCDC105 (CCDC105,57kDa)　Sperm样品
WT KO
180
130
95
70
55
43
33
Tubulin
WT KO
180
130
95
70
55
43
33
WT KO
CCDC105

Fig 5C

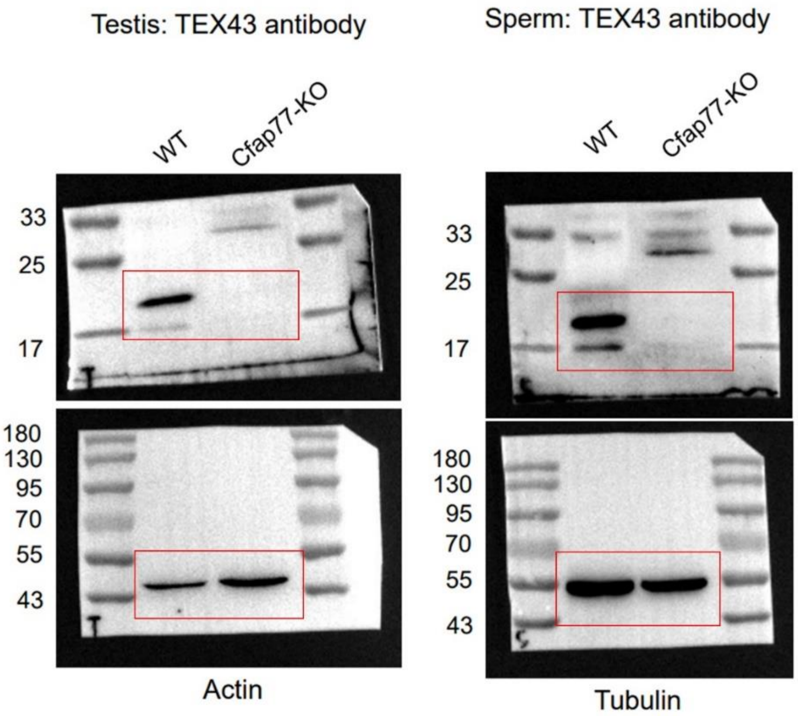
Testis: TEX43 antibody
Sperm: TEX43 antibody
WT
Cfap77-KO
WT
Cfap77-KO
33
25
17
33
25
17
180
130
95
70
55
43
180
130
95
70
55
43
Actin
Tubulin

S1A Fig

FLAG-CCDC105和HA-CFAP77互作

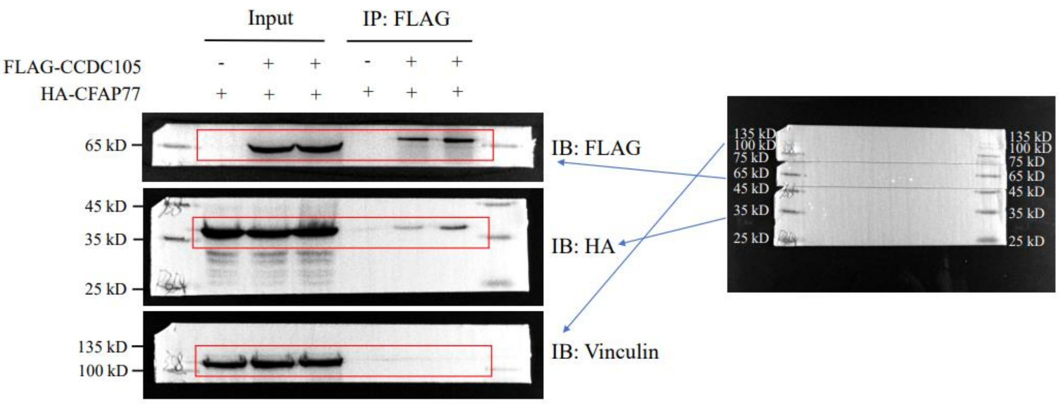

S1B Fig

FLAG-CCDC105和HA-TEX43互作

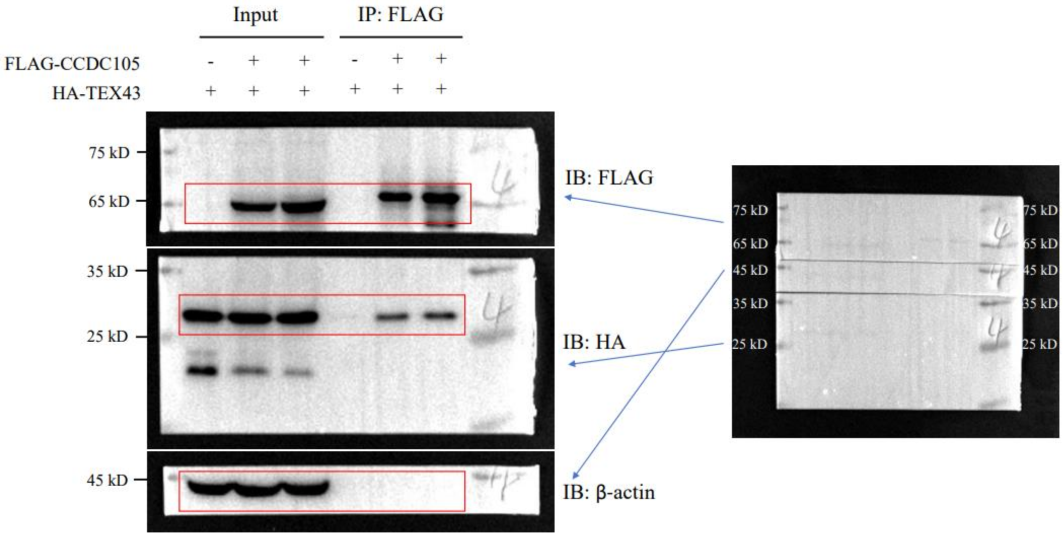

S1C Fig

MYC-CFAP77和FLAG-TEX43互作

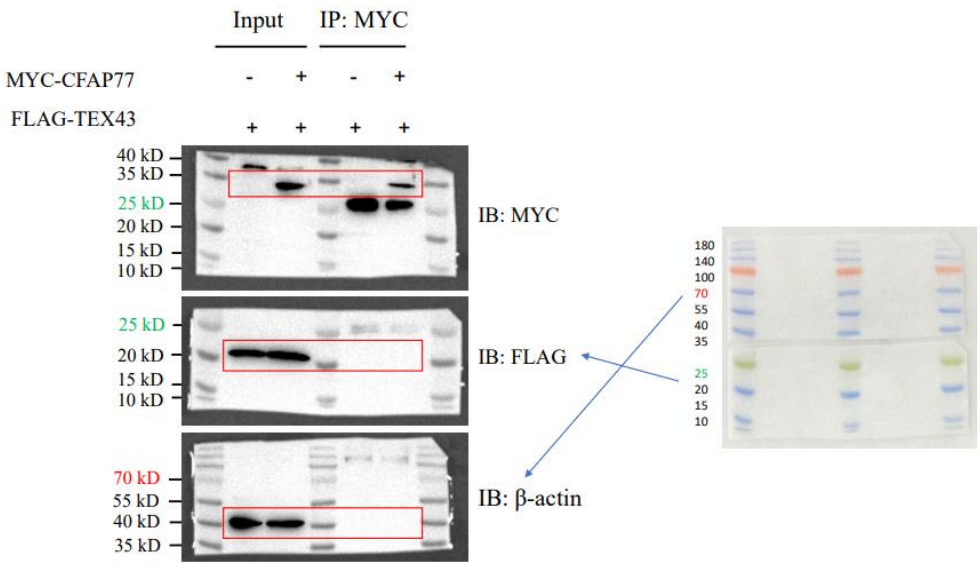

S10A Fig

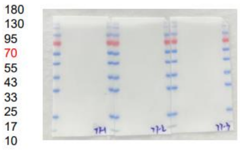

过表达验证抗体 （overexpression verification） - CFAP77

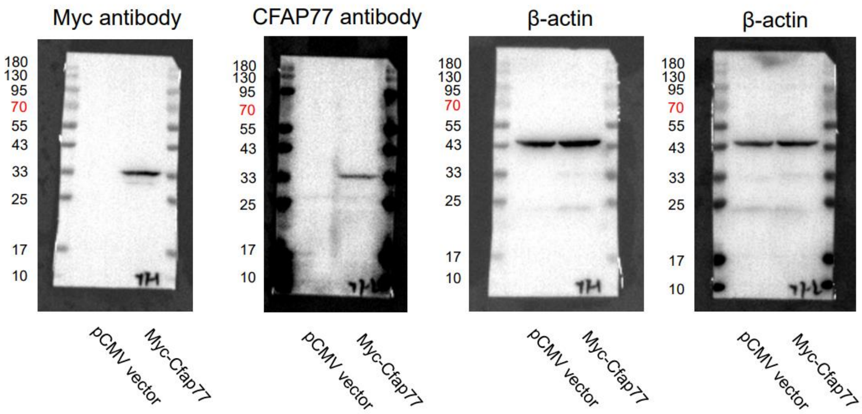

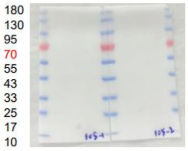

过表达验证抗体 （overexpression verification） - CCDC105

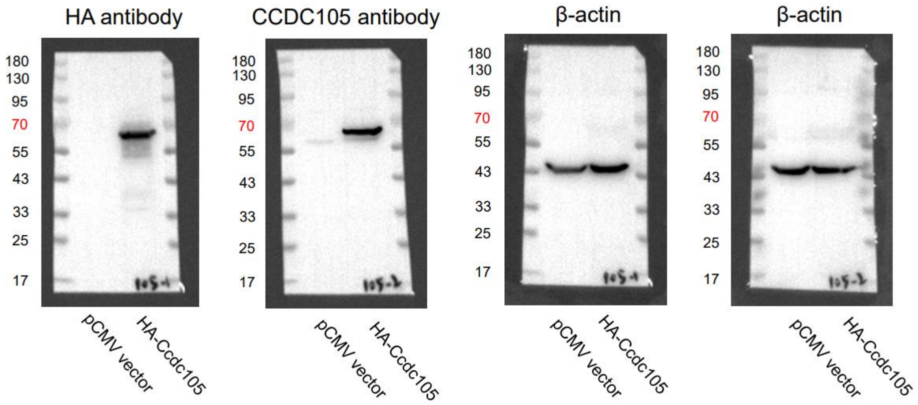

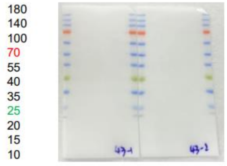

过表达验证抗体 （overexpression verification） - TEX43

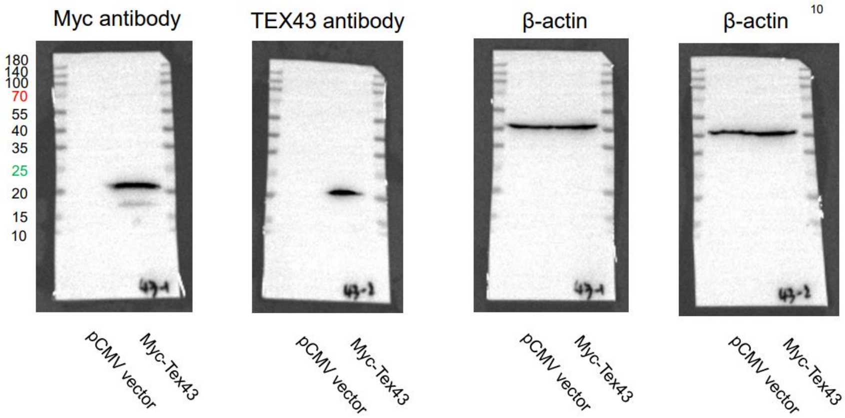

S10B Fig

敲除验证抗体 （knockout verification）- CFAP77

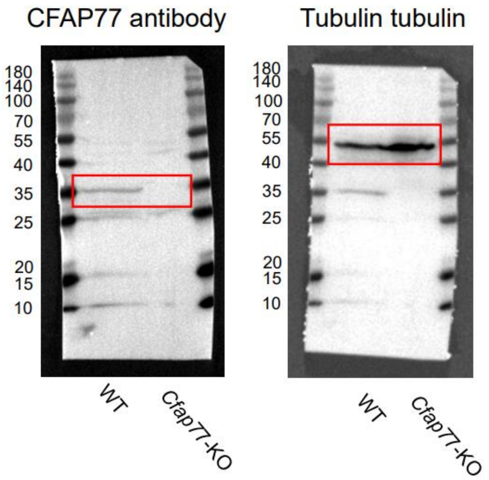

stripping之后杂tubulin antibody

S10C Fig

敲低验证抗体 （knockdown verification） - CCDC105

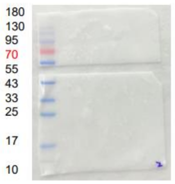

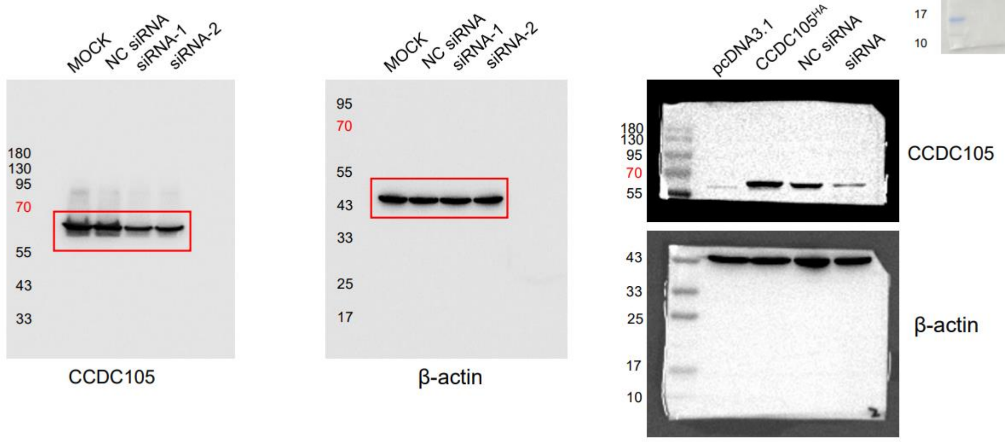

敲低验证抗体 （knockdown verification） - TEX43

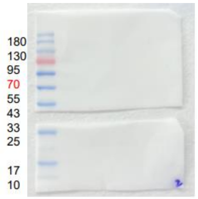

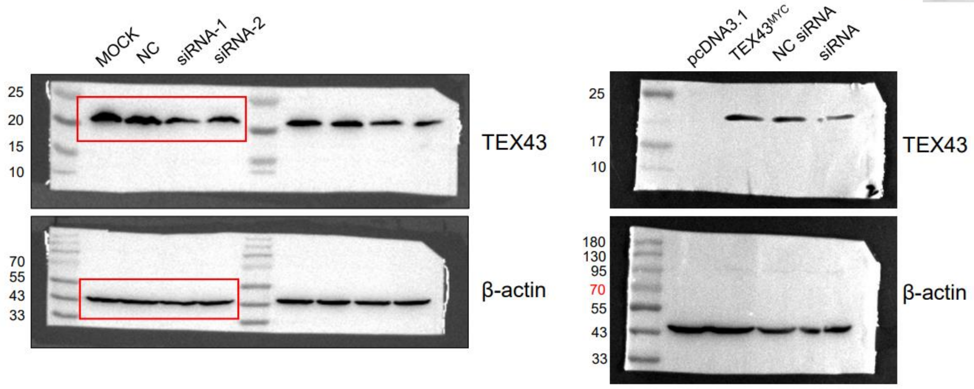

Supplement: S1 Raw Images — (PDF) [file pbio.3003442.s017.pdf]
