# Supplementary material for: The core outer junction protein CFAP77 connects A- and B-tubules within doublet microtubules of cilia and flagella
Source: PLoS Biol. 2025 Oct 21;23(10):e3003442. doi: 10.1371/journal.pbio.3003442 (PMC12551952; doi:10.1371/journal.pbio.3003442)
Supplement: Attachment — Submitted filename: Response to Reviewers.docx [file pbio.3003442.s020.docx]

**Rev. 1:**

Xia, Yin and colleagues address the importance of CFAP77 for doublet microtubule stability in mouse sperm. They demonstrate that genetic loss of CFAP77 causes sperm motility defects, male infertility, and B tubules open at the outer junction. They apply *in-situ* cryo-ET to show that loss of CFAP77 also causes loss of CCDC105 and TEX43. Overall, this is a convincing study with valuable findings that deserves publication once the following issues are resolved:

Thank you for your comments. We have carefully revised the manuscript according to your constructive suggestions.

1. The fertility measurements are statistically underpowered (*n*=3). Recommendations are that at least 4 litters are needed to provide reliable and robust estimates of fertility.

During the process of review and revision, additional pairs of WT and *Cfap77*-KO mice (*n*_male_=5 each now) were included to the fertility test. However, the degree of male infertility in *Cfap77*-KO mice remained unchanged. We have revised Fig. 2C and the corresponding position in the text.

2. The multiple mentions that it is a novel approach or that this the first study to combine in situ structural biology and knockout mice is incorrect and unnecessary.

We agree with the reviewer and have removed all exaggerated statements in the revised manuscript.

3. The section on CFAP47 in the introduction is an unnecessary distraction (in addition to countering their assertion that this paper is the first to combine cryo-ET and knockout mice).

Thank you for your suggestion. In the revised manuscript, we provide a short and focused introduction and the description of ‘cryo-ET + knockout mice’ has been deleted.

4. The failure of the cryo-ET approach to resolve classes in which the OJ is "open" suggests that the processing strategy may have been suboptimal and biased towards identifying those particles with a conventional doublet microtubule architecture. If a different strategy is used (e.g. initial processing ignoring the B tubule, followed by classification on the B tubule alone), can the authors recover additional states more consistent with the TEM data?

We thank the reviewer for this valuable suggestion. Following your advice, we reprocessed the cryo-ET data with two strategies: (1) We first applied a soft mask that enclosed only the A-tubule and ignored density from the B-tubule during initial alignment for all the DMT particles. We then applied a soft mask that enclosed only the B-tubule for 3D classification. This process yielded three distinct classes. Approximately 37 % of the particles adopted the canonical DMT architecture. One class (∼12%) displayed a clear “OJ-open” configuration that closely resembles the open DMT seen in our TEM data (new Fig. 4C). (2) We aligned the good DMT particles to the consensus map and applied a small mask around the OJ to perform 3D classification. Two additional subclasses emerged that show localized weakening of protofilament contacts: A10–B01 weak connections (∼20%) and B01–B02 weak connections (∼18%). Together, these make up ~38% of the good DMT dataset, indicating that many DMTs that appear globally intact despite harbouring incipient breaks at the OJ (new Fig. 4D). All new maps, particle distributions and the corresponding discussion have been incorporated into the revised manuscript.

5. Whether SPACA9/EUKUR/TEKT5 are absent or not from the *Cfap77*-KO doublet microtubules is not clearly addressed in the Results. A relatively low resolution 8-nm map may be insufficient to identify all the structural changes.

We agree with the reviewer that it is currently not possible to make an accurate determination of their absence. At present, the *Cfap77*-KO DMT particles can only produce an overall lower-resolution reconstruction with an 8-nm repeat (our reclassification attempts aimed at obtaining maps with longer repeats were unsuccessful). Since SPACA9, ENKUR, and TEKT5 are nonfilamentous proteins, their densities tend to blur easily when the average interval does not match their true periodicity. To avoid any confusion, we have deleted the description related to SPACA9/ENKUR/TEKT5 (this is also not the focus of our current study). Thank you for your understanding.

6. Why does the mass spectrometry volcano plot (Fig. 5) have no data points between +1 and -1 log2 (fold change)? All statistically significant changes should be labeled (especially as there are so few).

Thank you for pointing this out. This occurred because the data between +1 and -1 log2FC were not selected before the volcano plot was constructed. In the revised manuscript, we have redrawn the volcano plot. A cut-off point of a 2-fold change and a *p*-value<0.01 were selected as the screening criteria. The name of the proteins with significant changes are labelled. Moreover, all the differentially expressed proteins (2-fold changes with *p*-value<0.05) are listed in Table S3, and the MS data were deposited to ProteomeXchange (PXD056128).

7. It is unclear how the difference map was created. Ideally, it should be generated by subtracting reconstructions of WT and *Cfap77*-KO from similar numbers of particles at similar resolutions and with the same periodicities.

Thank you for your valuable feedback. In response, we have provided a detailed account of the process. We downloaded the WT map with a 16-nm repeat from the EMDB (EMD-35210/35211) and generated the 8-nm repeat WT map. Prior to making any comparisons, we resampled this map onto the same grid as our *Cfap77*-KO reconstruction (6.8 Å/pixel) and applied a low-pass filter to reduce the resolution to 24 Å. This preprocessing is a standard practice in cryo-EM difference mapping (see also PMID: 40473901). With respect to the concern about the number of particles, we acknowledge that the quality of particles can vary significantly across different datasets, particularly between WT and gene knockout samples. Consequently, achieving similar particle counts that also yield comparable resolutions and periodicities presents a considerable challenge.

8. Few details are provided as to how the TEM data were quantified. Was this analysis blind to genotype?

In this study, all tests were performed and analysed by an experimenter who was blinded to the genotype. Sperm from the four groups (*n*=3 each) were prepared for TEM analysis. For each mouse, 50 axonemes were randomly selected to examine the status of A-B tubule connections.

9. The manuscript should include a table of cryo-ET data collection and processing statistics, following the convention in the field.

Thank you for your suggestion. We added a table of cryo-ET data collection and processing (new Table S4).

Table S4. Cryo-ET data collection and data processing

| **Data acquisition** | |
| --- | --- |
| Microscope | Titan Krios G2 |
| Voltage (kV) | 300 |
| Detector | Gatan K2 |
| Energy filter | Gatan GIF Quantum, 20 eV |
| Mode | Super resolution |
| Pixel size (Å) | 3.4 |
| Stage tilting angle | -50° - 67° or -66° - 51° |
| Number of images | 39 or 40 |
| Exposure per image (e/Å²) | 3 or 3.5 |
| Exposure per tilt (e/Å²) | 117 to 140 |
| Defocus range (μm) | -1 - -4 |
| Software | SerialEM |
| **Sub tomogram analysis** | |
| Software | RELION-3.1/Warp |
| EMDB ID entry  (composite map) | EMD-63176 |
| Number of tomograms | 100 |
| Number of particles extracted | 65776 |
| Number of final particles | 10020 |
| Symmetry | C1 |
| Resolution (Å) | 24 |
| Map pixel size (Å) | 6.8 |

10. In the TEM images, is it possible to quantify if the open B tubules still have 10 protofilaments (as drawn in the proposed model)?

This is an interesting question. Directly determining the number of protofilaments via traditional TEM analysis is impossible. As shown in response 4, we obtained DMT maps with different degrees of OJ openness through two classification strategies (Fig 4 C and D). These maps show that the B-tubules are all composed of 10 protofilaments.

11. The phylogenetic distribution of CFAP77 should be addressed more clearly. For example, two recent structural studies have shown that CFAP77 homologs are not present in trypanosomatids.

Thank you for reminding us of these two recent interesting studies. *Trypanosoma brucei* diverges early within the eukaryotic lineage before the emergence of separate lineages for humans/mice and other organisms (e.g., *Chlamydomonas* and *Tetrahymena*) used as models for cilium biology. We have added the following description: ‘CFAP77 orthologues are not present in the axoneme of *Trypanosoma brucei* (Doran MH et al., Science, 2025; Xia X et al., Science, 2025), indicating that the mechanism underlying OJ maintenance may differ between flagellated parasites and other commonly-used eukaryotic ciliary organisms’. We have also revised Fig. 6A to clearly indicate the absence of CFAP77 homologues in *Trypanosoma brucei*.

Minor comments.

1. Past and present tenses are used inconsistently in the Methods and figure legends, e.g. in the Data Processing section and Figure 1.

Thank you. We corrected the tenses and polished the manuscript by the editing services of Springer Nature.

2. Please provide more sample preparation details for the "Quantitative proteomics" section. For example, how many sperm cells were used?

Sperm were retrieved from the cauda epididymis of 10-week-old *Cfap77*-KO mice and WT mice (*n*=3 per group), and 1×10^7^ sperm from each group were used for quantification proteomics.

3. The acronym SEM is used to mean both scanning electron microscopy and standard error.

Thank you. We use scanning electron microscopy (not the acronym) in the revised manuscript.

4. The Discussion should clearly state that the evidence shows CFAP77 is not necessary for B tubule formation but only for its stability.

We have added the following paragraph: ‘B-tubules nucleate at the surface of A-tubules; however, the molecular mechanism governing B-tubule formation is largely unclear. An *in vitro* DMT assembly study suggested that removal of the C-terminal tail of A10 and A11 protofilaments allows the nucleation of a B-tubule from the B01 protofilament (Schmidt-Cernohorska M et al., Science, 2019). In *Cfap77*-KO mice, open B-tubules specifically at the OJ region were observed only in approximately half of the DMTs, whereas many A- and B-tubules remained intact. Our study indicates that CFAP77 is not necessary for B-tubule formation but only for the stability of the OJ.’

5. L48. It is the dynein tails that associate with the A tubule, and the heads that associate with the neighboring B tubule. The text currently has the order wrong. It is usual to refer to the N-DRC as having a "tail".

Thank you. We have revised this sentence to read ‘DMTs are docked with a set of axonemal complexes, such as outer and inner dynein arms (ODAs and IDAs), radial spokes and a nexin-dynein regulatory complex (N-DRC)’.

6. L54. To be inclusive and comprehensive, studies of bovine sperm structure should be added.

We added important studies of bovine sperm DMTs (Leung MR, et al. Cell. 2023; Leung MR, et al. Nature. 2025).

7. L57. It should be made clear that CFAP77 is not the only protein present at the OJ, connecting A and B tubules.

Thank you. We have revised this phrasing to read ‘CFAP77 and other proteins are localized at the OJ regions’.

8. L65. "Understating" should be "understanding"

This paragraph has been deleted in the revised manuscript.

9. L74. "knockout mice were male infertile" should be "male knockout mice were infertile"

Thank you. We have used ‘male knockout mice were infertile’ as suggested.

10. L98. "glutarnine" should be "glutamine"

We have corrected the spelling mistake.

11. L112. Unnecessary to mention this is the "first" *Cfap77*-KO mouse strain.

We have deleted the term ‘first’ throughout the manuscript.

12. L137. "focusing on axoneme" should be "focusing on the axoneme"

As suggested, we have added ‘the’.

13. L155. All instances of "perfectly consistent" should be replaced with "consistent".

Thank you. We have deleted ‘perfectly’.

14. L156. What is meant by "expression evidence"? Structural data from WT doublet microtubules seems more appropriate to reference.

We have used ‘structural data from WT DMTs’ as suggested.

15. L159. "frozen" should be "froze"

We have corrected this mistake.

16. L168. "This sperm DMTs" should be "The sperm DMTs"

Thank you. We have corrected this mistake.

17. Fig. 1. It would be helpful to show a zoomed in cross-sectional view of the OJ, sliced appropriately to see the arrangement of CFAP77/CCDC105/TEX43.

As suggested, we have added a zoomed-in cross-sectional view of the OJ (new Fig. 1B).

18. Fig. 1. In the zoomed-in panels, it would be helpful to show the interacting residues, e.g. P110-H114, in a slightly different shade of color. Stick representation could be used to show the residues mutated in panels E and F.

Following your advice, we have modified the colour of the amino acids and provided sticks to indicate the residues mutated in the co-IP experiments (new Fig 1C-E).

19. Fig. 5 "unchange" should be "unchanged"

We have corrected this in the revised Fig 5.

20. Fig. 6B. Please also provide zoomed-in images of doublet microtubules from WT mice tracheal cilia.

As suggested, we have provided a zoomed-in image of WT DMT in the revised Fig 6B.

21. Fig. 6C - the y axis shows percent rather than ratio.

Thank you. We have changed ‘ratio’ to ‘percentage’ in the revised Fig. 6C.

22. Fig. S4. Please give extract particle numbers.

We have provided information on cryo-ET data collection and data processing (new Table S4).

23. Fig. S6 A. The color-coding of the multiple-sequence alignment is subjective. The alignment might also benefit from additional species.

As suggested, we chose more commonly-used eukaryotic ciliary organisms (humans, bovines, mice, sea urchins, *Tetrahymena*, and *Chlamydomonas*) and rebuilt a multiple-sequence alignment map using the ClustalW and ESPript websites.

24. Fig. S6 B. Why show Alpha Fold models, when the experimentally determined structures could be compared?

Thank you. We have deleted Fig S6B.

25. Please combine the two supplementary movies so that they are side-by-side. This facilitates comparison of the phenotypes.

We have combined the two supplementary movies in the revised manuscript.

**Rev. 2:**

The authors Xia et al., are studying poorly characterized gene/protein Cfap77 and demonstrate its critical role in murine fertility by uncovering the mechanism of its action by using a knock-out mouse model and state-of-the-art Cryo-electron tomography.

-The research question is clearly formulated, the experiments are specific and well-designed: e.g., mutating specific a.a. in Cfap77 instead of deleting the entire gene to validate the interactions in the ternary subcomplex (CFAP77-CCDC105-TEX43).

-The localization of Cfap77 to the microtubular doublet at the outer junction of A and B tubules was already reported earlier (in Tetrahymena https://doi.org/10.1038/s41467-023-37868-0, see urchin, bovine https://doi.org/10.1016/j.cell.2023.05.026); however, its phenotype and function mechanism in mammals is not known.

-In a nutshell, experiments have logical flow and validation with included controls, they demonstrate Cfap77 central role in male fertility in association with the loss of three proteins (CFAP77-CCDC105-TEX43) from the DMT outer junction that way providing the functional mechanism. All in all, technically study is sound and provides novel insights into the previously unreported function of the Cfap77 gene. However, the study would benefit from data representation and discussion polishing and language revision.

Thank you for your comments. We have carefully revised the manuscript according to your constructive suggestions.

Some specific points to consider:

1. IVF was done only with the cumulus-intact oocytes. To confirm if fertility reduction is associated with motility aberrations or sperm abnormalities, cumulus-free and Zona pellucida-free IVF should be done. It is likely that ZP-free oocytes could be fertilized in as wild types which would be beneficial information when planning ART for human cases.

Thank you for your valuable suggestions. Cumulus-free IVF and ZP-free IVF were added to the revised manuscript. Cumulus cells were removed by treatment with 350 µg/mL hyaluronidase for 10 min. To eliminate the zona pellucida, the oocytes were treated with 1 mg/mL collagenase for 8 min. The capacitated sperm were introduced into a drop containing cumulus-intact, cumulus-free, or zona pellucida-free oocytes at 37°C under 5% CO_2_. Final concentrations of 2×10^6^ sperm/mL and 2×10^5^ sperm/mL were used for cumulus-intact/free IVF and zona pellucida-free IVF, respectively. We revised the manuscript as follows: ‘*In vitro* fertilization (IVF) assays using cumulus-intact oocytes (Fig 2D) or cumulus-free oocytes (Fig 2E) revealed that the percentage of 2-cell embryos was significantly lower in the groups with sperm from *Cfap77*-KO mice than in those with sperm from WT mice. However, the percentage of 2-cell embryos generated from *Cfap77*-KO sperm with ZP-free oocytes was comparable to that of the control (Fig 2F), indicating that CFAP77 loss does not affect sperm-egg fusion or genomic integrity’.

2. Line 129: 'Collectively, the animal studies indicate that CFAP77 is critical for progressive sperm motility and male fertility in mammals.' This is likely too bold a statement since data is only from the mouse, given this, it only suggests importance in mammals but does not prove.

Thank you for pointing this out. We have revised this sentence as follows: ‘Collectively, these findings indicate that CFAP77 is critical for progressive sperm motility and male fertility in mice.’

3. Researchers analyzed duplet microtubules with open B microtubules in *Cfap77*-KO in ependymal, tracheal, and nasal cilia. It would be useful to show the expression levels of Cfap77 by RT-qPCR or Western blot, at least in these tissues, or even a broader selection of ciliated tissues in comparison to testes and epididymis, where phenotype is prominent.

As suggested, we examined the mRNA level of *Cfap77* in 11 mouse tissues by qRT‒PCR in the revised manuscript. *Cfap77* mRNA was highly expressed in the epididymis, testis, and ependyma but was expressed at low levels in other tissues (revised Fig. S7D).

4. Line 236: 'A combination of knockout animal studies with structural biology technologies is one of the cutting-edge trends in cilia biology: functional studies are essential to confirm the physiological roles of axonemal proteins and structural biology can further provide in-cell structural insight of how knocking out a protein disrupts the axoneme ultrastructure at near-atomic level.' Requires citations.

Thank you. We have provided the key reference (Chen Z et al., Cell, 2023; PMID: 37865089).

5. Line 243: 'Our current study represents a good example to combine structural biology and knockout animals.' Liguistuly not sound.

Thank you. We have revised this sentence as follows: ‘Our current study further illustrates the advantages of the combination of structural biology and knockout animal experiments.’

6. Line 247: 'Moreover, we also found that SPACA9 and ENKUR were missing in the B-tubule, and tilted Tektin5 (Tektin5-5) protein was absent in A-tubule lumen of sperm DMTs from Cfap77-KO mice.' This sentence refers to results that are not presented in the results section and are out of context as such.

We apologize for the misunderstanding. At present, the *Cfap77*-KO DMT particles can only produce an overall lower-resolution reconstruction with an 8-nm repeat. Our reclassification attempts aimed at obtaining longer repeats were unsuccessful. Since SPACA9, ENKUR, and TEKT5 are nonfilamentous proteins, their densities tend to blur easily when the average interval does not match their true periodicity. To avoid any confusion, we have deleted descriptions related to SPACA9, ENKUR, and TEKT5. This is also not the focus of our current study.

7. Line: 'From the perspective of the current study, we consider that CFAP77 is the evolutionarily conserved core OJ protein residing between A11-A12 and B01-B02, while CCDC105 and TEX43, two sperm-specific MIPs, may function to join CFAP77 molecules into a more secure footing for DMTs A-B tubule connection in sperm flagella.' In other parts of the text, authors discuss CFAP77 as an evolutionarily conserved protein, and here they write that 'we consider that CFAP77 is the evolutionarily conserved', why just 'consider'?

Thank you. We have clarified this expression and revised this sentence as follows: ‘CFAP77 is an evolutionarily conserved core OJ protein, whereas CCDC105 and TEX43 are two sperm-specific MIPs.’

8. How was the specificity of in-house derived antibodies validated (anti-CFAP77, mouse anti-TEX43)?

As suggested, we performed overexpression (OE) and knockout (KO)/knockdown (KD) antibody validation. For OE validation, HEK293T cells were transfected with *Cfap77*-, *Ccdc105*-, or *Tex43*-tagged plasmids. We found that both tag-antibodies and their antibodies could recognize a specific band of the same molecular size (new Fig. S8A). For KO validation, we showed that CFAP77 was absent in sperm samples of *Cfap77*-KO mice (new Fig. S8B). For KD validation, we found that siRNAs targeting *Ccdc105* and *Tex43* reduced their signals in HEK293T cells transfected with *Ccdc105*- or *Tex43*-tagged plasmids, respectively (new Fig. S8C). The Western blotting data (Fig. 5B and C) were also consistent with the *in situ* DMT analysis (Fig. 4) and sperm proteomics (Fig. 5A) in *Cfap77*-KO mice, showing the (near) complete absence of CCDC105 and TEX43 after the loss of CFAP77.

9. Line 402: cDNA encoding CFAP77, CCDC105, and TEX43 Italic.

Thank you. We have corrected the mistake as suggested.

**Rev. 3:**

In their study Xia et al. investigate the role of the outer junction CFAP77-CCDC105-TEX43 complex, previously identified in their cryo-EM analysis (Tai et al., 2023). Through alanine mutagenesis and co-IP experiments, the authors pinpointed key residues essential for complex formation. They generated a *Cfap77*-KO mouse model and demonstrated that the mice are infertile due to impaired sperm motility, likely resulting from disrupted outer junction architecture, as shown by electron microscopy. Cryo-EM/ET and mass spectrometry analyses confirmed that loss of CFAP77 leads to the absence of CCDC105 and TEX43. Interestingly, although CFAP77 is also expressed in other tissues where it similarly induces an open outer junction, no additional phenotypes were observed.

Overall, the manuscript is well written, and the narrative flows logically. The study presents compelling in vivo evidence that the stability of the outer junction structure is mediated by a defined protein complex, with CFAP77 as a central component. These findings provide significant insight into the molecular determinants of axonemal stability by linking specific structural components to ultrastructural integrity and motility.

Thank you for your comments. We have carefully revised the manuscript according to your constructive suggestions.

1. My main concern lies with the statistical reporting. In several key fertility-related assays, including litter size, IVF, and sperm count/motility, the use of *n*=3 biological replicates appears insufficient to support the conclusions with statistical confidence. I strongly recommend increasing the number of animals analyzed or, at the very least, providing a clear rationale for the chosen sample sizes. Additionally, the number of cells/sperm assessed should be explicitly stated (Fig. 2E, H, I). An *n* of 3 in these assays limits the power of the comparisons and may compromise the robustness of the conclusions.

Thank you for your concerns. During the process of review and revision, more pairs of WT and *Cfap77*-KO mice were added to the fertility test (*n*=5 each), cumulus-intact IVF (*n*=5 each), cumulus-free IVF (*n*=5 each), sperm count (*n*=5 each), and progressive motility (*n*=5 each). We have revised Fig. 2C-J accordingly. In the revised figure legend, we clearly indicate the concentration/number of sperm assessed. For sperm motility, three fields of view were randomly selected and at least 200 sperm from each sample were analysed. The capacitated sperm were introduced into a drop containing cumulus-intact or cumulus-free eggs at a final concentration of 2×10^6^ sperm/mL.

2. I also have concerns regarding the use of homemade primary antibodies. Specifically, the predicted molecular weight of CCDC105 is approximately 50 kDa, which raises the possibility that the lower band in Figure 5B corresponds to CCDC105, rather than the upper band as currently indicated. While this point requires clarification, the mass spectrometry data does provide complementary support for the author’s findings.

Thank you for your question. We validated the overexpression (OE) and knockout (KO)/knockdown (KD) antibodies. For OE validation, HEK293T cells were transfected with *Cfap77*-, *Ccdc105*-, or *Tex43*-tagged plasmids. We found that both the tag-antibodies and their antibodies recognized a specific band of the same molecular size (new Fig. S8A). For KO validation, we showed that CFAP77 was absent in sperm samples of *Cfap77*-KO mice (new Fig. S8B). For KD validation, we found that siRNAs targeting *Ccdc105* and *Tex43* reduced their signals in HEK293T cells transfected with *Ccdc105*- or *Tex43*-tagged plasmids, respectively (new Fig. S8C). The Western blotting data (Fig. 5B and C) were also consistent with the *in situ* DMT analysis (Fig. 4) and sperm proteomics (Fig. 5A) in *Cfap77*-KO mice, which revealed the (near) complete absence of CCDC105 and TEX43 after the loss of CFAP77.

Minor Comments:

1. It would be beneficial to include co-IP experiments with TEX43 to investigate protein interactions and to strengthen the authors claim.

As suggested, we added co-IP experiments involving CCDC105-TEX43 and CFAP77-TEX43 to the revised manuscript. HA-tagged TEX43 was immunoprecipited by FLAG-tagged CCDC105 using the FLAG antibody (new Fig. S9B). Furthermore, no interaction was detected between tagged-CFAP77 and TEX43 (new Fig. S9C). These co-IP results are consistent with the structural data (PDB: 8IYJ and 8I7R) showing that CCDC105 interacts with CFAP77 and TEX43.

2. It would be interesting to discuss why certain DMTs are more prone to structural defects than others.

As suggested, we have added a short discussion as follows: ‘We found that the disconnection of A- and B-tubules was distributed mainly in DMTs 1, 4, 5, 6, and 9 in the sperm axonemes of *Cfap77*-KO mice. However, why certain DMTs are more prone to structural defects than others is still unknown. The asymmetric architecture of mammalian sperm axonemes has been revealed by *in situ* cryo-ET (Chen Z et al., Nat Struct Mol Biol, 2023). Each of the nine DMTs is decorated with a distinct combination of complexes, and uneven force distribution occurs during sliding. We suggest that DMTs 1, 4, 5, 6, and 9 in axonemes may suffer excessive force during beating and are more prone to break at weak OJ sites after the loss of CFAP77; however, further experiments are needed to clarify this’.

3. Abstract + title: The authors use "DMT" as the abbreviation for "doublet microtubules" throughout the manuscript. To ensure consistency, they should revise the title and abstract to use the term "doublet microtubules" instead of "microtubule doublets."

Thank you. We have changed all instances of ‘microtubule doublets’ to ‘doublet microtubules’ throughout the revised manuscript.

4. Usually hyphen is used between A/B tubule → "A-tubule" and "B-tubule".

We have substituted ‘A-tubules’ and ‘B-tubules’ for ‘A tubules’ and ‘B tubules’ in the revised manuscript.

5. In their Intro the authors mention that CFAP20 is found in C. elegans (and zebrafish). It would be helpful to clarify that CFAP20 is also present in non-motile cilia, as C. elegans lack motile cilia.

Thank you. We have revised this sentence as follows: ‘CFAP20 has been reported to maintain the structural integrity of the IJ and is required for cilia motility in zebrafish as well as nonmotile cilia function in *C. elegans*’.

6. Page 5, Line 97: The phrase "relatively independent subcomplex" should be clarified. Specifically, do the authors mean that this complex does not physically or functionally interact with other microtubule inner proteins (MIPs)?

We apologize for the confusion. We have deleted the description of the ‘relatively independent subcomplex’ in the revised version.

7. Figure 5: It would be helpful if the authors could include information about proteins that were upregulated following Cfap77 knockout.

In the revised manuscript, we have redrawn the volcano plot. A cut-off point of a 2-fold change and a *p*-value<0.01 were selected as the screening criteria. The names of the proteins with significant changes are labelled. Additionally, all the differentially expressed proteins (2-fold changes with *p*-values<0.05) are listed in Table S3, and the MS data have been deposited to ProteomeXchange (PXD056128).
