# Supplementary material for: The core outer junction protein CFAP77 connects A- and B-tubules within doublet microtubules of cilia and flagella
Source: PLoS Biol. 2025 Oct 21;23(10):e3003442. doi: 10.1371/journal.pbio.3003442 (PMC12551952; doi:10.1371/journal.pbio.3003442)
Supplement: Attachment — Submitted filename: Response_to_Reviewers_auresp_3.docx [file pbio.3003442.s021.docx]

**Rev. 1:**

The authors have addressed all my concerns. The revised manuscript is clearly written and ready for publication. During reading I noticed only two sentences were the authors might want to consider changes: L26. Please remove "core" - it is unnecessary and potentially misleading as CFAP77 is not found in all doublet microtubules. L55. "explored" should be "determined".

Thank you for your positive comments. As suggested, we removed “core” in L26 and used “determined” in L55.

**Rev. 2:**

I have reviewed the response to my criticism and can conclude that it was appropriately addressed. However, the response letter contained incorrect information about the actual image numbers in the manuscript versus revised text: e.g. S7D->S8D; new Fig. S8B->S10B. After revising the manuscript text, it seems to be sound.

Thank you for pointing out the mislabeling in the “response to reviewers” (the S7D should be S8D and the S8B should be S10B). We confirm that the text in the manuscript is correct.

**Rev. 3:**

The authors have addressed all my concerns. The manuscript has improved significantly and I recommend it for publication in PLOS Biology.

Thank you for your positive comments.
